# *Mycobacterium ulcerans* Infection Imported from Australia to Missouri, USA, 2012

**DOI:** 10.3201/eid2011.131534

**Published:** 2014-11

**Authors:** Benjamin Stuart Thomas, Thomas C. Bailey, Julu Bhatnagar, Jana M. Ritter, Brian D. Emery, Omar W. Jassim, Ian Kerst Hornstra, Sarah L. George

**Affiliations:** Washington University School of Medicine, St. Louis, Missouri, USA (B.S. Thomas, T.C. Bailey, I.K. Hornstra);; Centers for Disease Control and Prevention, Atlanta, Georgia, USA (J. Bhatnagar, J.M. Ritter, B.D. Emery);; John Cochran Veterans Affairs Medical Center, St. Louis (O.W. Jassim, I.K. Hornstra, S.L. George);; Saint Louis University, St. Louis (S.L. George)

**Keywords:** Buruli ulcer, Mycobacterium ulcerans, oral regimen, Daintree ulcer, Australia, United States, skin ulcers, bacteria, acid-fast bacilli, imported infection

## Abstract

Buruli ulcer, the third most common mycobacterial disease worldwide, rarely affects travelers and is uncommon in the United States. We report a travel-associated case imported from Australia and review 3 previous cases diagnosed and treated in the United States. The differential diagnoses for unusual chronic cutaneous ulcers and those nonresponsive to conventional therapy should include *Mycobacterium ulcerans* infection.

Buruli ulcer disease, caused by *Mycobacterium ulcerans*, is the third most common mycobacterial disease worldwide. Cases are concentrated in sub-Saharan Africa but also occur in subtropical and nontropical regions (e.g., Australia and Japan) (*1*). *M. ulcerans* disease is not endemic to the United States, but rare cases have been reported in travelers returning from regions where the organism is endemic (*2*–*4*). We describe *M. ulcerans* infection in a man who returned to the United States after living abroad and review *M. ulcerans* cases reported in 3 travelers.

## The Case

In December 2012, after unsuccessful treatment elsewhere, a 63-year-old white man was referred to the Veterans Affairs Medical Center, St. Louis, Missouri, USA, for evaluation of skin ulcers. Previous medical history indicated hypertension and hyperlipidemia. The patient sought care in April 2012 for a persistent, nonpainful ulcer (1-cm diameter) on his right medial calf (Figure). No previous episodes or other symptoms of infection were reported. The patient had lived abroad for >10 years and had recently returned to the United States from Queensland, Australia, where he spent his last month hiking in the Daintree Rainforest. His health care providers recommended topical antimicrobial drugs and wound care, and a punch biopsy was obtained but the specimen was not stained or cultured for acid-fast bacilli (AFB). Pathologic findings on the biopsy specimen were nonspecific (reactive epidermal changes and ulceration).

**Figure:**
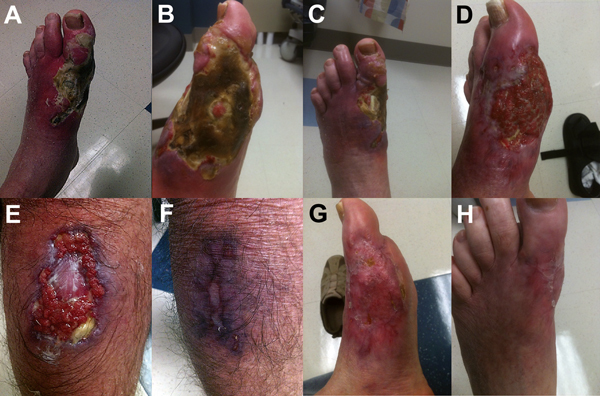
Progression of lesions caused by *Mycobacterium ulcerans* infection before, during, and after treatment. A–C) Left foot before treatment. D) Left lower leg during treatment. E) Right calf before treatment. F) Right calf after treatment. G, H) Left foot after treatment.

Between April and July 2012, the ulcer increased to 4 cm despite multiple debridements. In September a painful, warm, erythematous lesion developed on the patient’s left great toe. He consulted physicians in numerous subspecialties and underwent 2 more biopsies; specimens were not initially stained or cultured for AFB. Pathologic findings from the specimens showed extensive necrosis without granulomas. The ulcers were diagnosed as pyoderma gangrenosum with bacterial superinfection, and treatment was initiated with oral antimicrobial drugs (amoxicillin/clavulanate 875/125 mg twice daily) and prednisone (60 mg/day). Transient improvement occurred, but in late November, the toe lesion worsened.

When the patient was referred to the Veteran’s Affairs Medical Center in December 2012, biopsies were again obtained; pathologic evaluation showed extensive necrosis (without granulomas) and numerous AFB. Fresh frozen tissue was sent to the Centers for Disease Control and Prevention (CDC; Atlanta, GA, USA) for mycobacterial testing. Empiric treatment with levofloxacin, doxycycline, and azithromycin was initiated for nontuberculous mycobacterial infection, and the previously prescribed prednisone was rapidly tapered and stopped. At CDC, immunohistochemical testing of tissue for mycobacteria showed extensive bacilli. DNA extracted from biopsy samples was evaluated by PCR targeting 16s rRNA and the IS*2402* insertion element (*5*). Sequences of IS*2404* amplicons were 100% identical with those for *Mycobacterium ulcerans*. Tissue cultured (30°C) at CDC grew AFB after 3 months (toe specimen) and 4 months (calf specimen); 16S rRNA gene sequencing confirmed a *Mycobacterium* species, most closely matching *M. marinum* or *M. ulcerans*.

Therapy, guided by World Health Organization recommendations (*1*), was changed to rifampin (900 mg/day or 10 mg/kg bodyweight), clarithromycin (1,000 mg/day), and moxifloxacin (400 mg/day). In February 2014, after 15 months’ of antimicrobial drug treatment, debridement, and skin grafting to the left foot, the lesions were completely healed.

## Conclusions

Imported *M. ulcerans* disease is exceedingly rare, even in today’s age of global travel. When the infection does occur, diagnosis is often delayed. In addition to the case reported here, 3 other cases imported to the United States have been reported in the literature since 1967 (*2*–*4*).

The 4 *M. ulcerans* cases diagnosed in the United States were in men (median age 35 years, range 20–63) (Table). Lesions were located on the upper (25%) and lower (75%) extremities, similar to cases in Africa, where lesions commonly develop on the extremities of adults and on the trunk, head, neck, and upper limbs of children (*6*). Specific exposures were not identified in 3 of the US patients; the fourth had exposure to fresh water, a known risk factor (*6*). The 2012 imported US case is similar to cases in Australia, where patients have a median age of 61 years at diagnosis, and most have single lesions (95%) involving lower limbs (61%). However, in Australia, the median time from symptom onset to diagnosis is 42 days, consistent with greater familiarity with the disease (*7*).

**Table:** Characteristics of persons with *Mycobacterium ulcerans* infection diagnosed and treated in the United States but acquired in a different country*

**Patient no., age, y**	Location of ulcer	Risk factor	Travel history	Time, wk, to first drug therapy	Final treatment regimen	Time, mo, to diagnosis	Length, mo, of drug therapy	Surgical management	Outcome
**1, 20**	Left foot	None	Nigeria	20	Lamprene, clofazimine	–	–	Amputation below knee	Amputation
**2, 34**	Right elbow	None	Nigeria	–	Surgery alone	7	–	Debridement and split-thickness skin grafting	Cure
**3, 36**	Left calf	Fresh Water	Northern, western, and Central Africa	17	Clarithromycinand ciprofloxacin	8	18	Debridement and split-thickness skin grafting	Cure
**4, 63**	Right calf and left foot	Hiking in Daintree Rainforest in sandals	QLD, Australia	36	Rifampin, clarithromycin, moxifloxacin	9	15	Debridement and split-thickness skin grafting	Cure

*All patients were male. Except for negative smear results for patient 1, culture and smear results for all patients were positive. QLD, Queensland; –, Information not available.

The 3 persons with the prior cases of imported *M. ulcerans* disease in the United States had traveled to western Africa; the case-patient described herein had returned from Australia. Most *M. ulcerans* cases in Australia are linked to temperate, coastal Victoria and tropical, northern Queensland (*8*). Persons with cases imported to other *M. ulcerans*–nonendemic countries mostly traveled to Africa (1 traveled to South America), where the disease is present but uncommon (*9*–*12*).

In regions where *M. ulcerans* disease is endemic, it is readily recognized on the basis of lesion appearance and chronicity. In areas of Australia where Buruli ulcer disease is nonendemic, diagnosis is delayed (*13*). A hallmark of imported cases is the difficulty in arriving at a diagnosis due to nonfamiliarity with the disease. For the 4 imported US cases, the median time to empiric antimycobacterial therapy was 20 weeks, and the median time to definitive diagnosis was 8 months. The differential diagnosis for *M. ulcerans* disease is broad, spanning infectious and noninfectious etiologies, including filariasis, phycomycosis, resolving furuncle, and pyoderma gangrenosum. Samples from the case-patients were uniformly AFB smear-positive, and for 1 case in which the culture failed to grow *M. ulcerans,* diagnosis was made by clinical and epidemiologic history and presence of AFB in tissue. Because the organism is slow growing, prolonged culture (>3 months) at low temperatures is required. A hallmark of *M. ulcerans* histopathology is the absence of granulomas and presence of extensive necrosis caused by the organism’s secretion of mycolactone toxin, which suppresses the host’s immune response (*7*). The absence of granulomas on hematoxylin and eosin staining may result in tissue not being stained for AFB unless clinicians have a high index of suspicion. Furthermore, depending on the bacterial load and focal distribution, bacteria may not be detected by AFB staining. Molecular analysis of tissue is the most sensitive and specific method for rapid and confirmatory diagnosis of *M. ulcerans* and should be pursued when disease is suspected (*14*).

Treatment of *M. ulcerans* disease has changed markedly over the last several decades. Prior to the early 2000s, availability of effective drugs was limited, so surgery was the primary treatment. However, combination therapy with streptomycin, rifampin, and surgery (including skin grafting) has been shown to be effective (*1*), and some cases can be managed with medical therapy alone. Standard antimicrobial treatment, according to World Health Organization guidelines, consists of administering rifampin (10 mg/kg body weight daily by mouth) for 8 weeks and streptomycin (15 mg/kg body weight daily by intramuscular injection) for 8 weeks (*1*). Extensive clinical experience has also shown a combination of oral antimicrobial drugs (rifampin with clarithromycin or moxifloxacin) to be effective against Buruli ulcer disease (*15*). Of the 4 reported US cases, 1 required surgery alone and 3 required drug treatment and surgery. Response to therapy has been favorable: 2 US cases were cured by treatment with oral antimicrobial drugs.

Because awareness of Buruli ulcer disease is limited in regions where *M. ulcerans* is nonendemic, the potential for delayed diagnosis in such areas is increased. For persons with recent travel from *M. ulcerans*–endemic regions, Buruli ulcer disease should be considered in the differential diagnoses of unusual chronic cutaneous ulcers and skin ulcers nonresponsive to conventional therapy.
